# Brain structure variability study in pilots based on VBM

**DOI:** 10.1371/journal.pone.0276957

**Published:** 2023-01-27

**Authors:** Kaijun Xu, Rui Liu, Xi Chen, Yong Yang, Quanchuan Wang

**Affiliations:** Institute of Flight Technology, Civil Aviation Flight Academy of China, Guanghan, Sichuan, China; Kochi University of Technology, JAPAN

## Abstract

The impact of occupations on brain structures has attracted considerable research interests in the last decade. The aim of this research is to find the effect of flight training on brain gray matter volume of pilots. The whole-brain structural magnetic resonance imaging (sMRI) data collected from 26 pilots and 24 controls was analyzed using Voxel-based morphological analysis method (VBM) combined with T1 data to quantitatively detect the local gray matter of brain tissue and calculate the gray matter volume. The result shows that the pilot group has larger gray matter volume in the lingual gyrus and fusiform gyrus compared to the control group (P<0.05). Furthermore, there is a positive correlation between the gray matter volume and the number of flight hours (r = 0.426, P = 0.048) after studying the average gray matter volume value of the agglomerate of participants whose flight hours are between 0 and 1000 hours. The lingual gyrus and fusiform gyrus are involved in high-level visual processing, memory, multisensory integration and perception. The study has indicated the flight training could enlarge gray matter volume in the lingual gyrus and fusiform gyrus. During flying, pilots need to observe the instrumentation in the cockpit and fully interpret the readings, which may lead to the results.

## Introduction

Flight training has been identified as one of essential task for flight safety, with the aim of high-quality development of civil aviation. Safety management in civil aviation of China is entering a stage of performance-based system safety management. Due to the rapid growth of total civil aviation transportation in China, safety risk pressure is increasing day by day. Inconsistency between the behavioral response–based flight training mechanism of airlines and the profound changes in the operating environment, is becoming increasingly prominent.

As pointed out in "Statistical Bulletin of Civil Aviation Industry Development in 2019" published by the Civil Aviation Administration of China, there were a total of 570 air transportation safety issues that year, of which 11 were serious. The rates of serious safety issues and human-caused reasons for liability per ten thousand hours were 0.009 and 0.023, respectively. A survey by the International Air Transport Association shows that 80–90% of flight accidents are caused by human factors. Human factors have been widely recognized as a key element to ensure aviation safety and efficiency. Therefore, measures to decrease the proportion of unsafe incidents arising from human causes, reduce the rate of accidents rate caused by crew, and promote the high-quality development of civil aviation are undoubtedly an important direction for flight training reform in the future. As the core part of the flight process, pilot occupation competency is even more important. Data-driven analysis of the variability of pilots’ brain structure can provide objective and quantitative indicators of their core competency in the professionalism life-cycle management (PLM) system, provide a theoretical basis for the neuroimaging based evaluation and selection of pilots, and improve civil aviation flight training and operational safety capabilities [1].

The cerebral cortex of the human brain plays a very important role in various functions in people’s daily lives. In recent years, brain science has been frequently studied. In 2013, the United States (http://www.braininitiative.nih.gov), Europe (https://www.humanbrainproject.eu) and other regions proposed brain research plans, and China officially launched the One Two Wing Brain Science Research Project in 2016 [2]. The rapid development of brain imaging technology will undoubtedly integrate the multi-level development of brain research molecules, cells, and macroscopic brain structure and functions [3]. Moreover, pilots’ brain function and brain structure are significantly different from those of ordinary occupation. There have already been a few studies applying functional magnetic resonance imaging (fMRI) to pilots in China. Liu et al. [4], studied the brain functional mechanisms of pilots during exposure to hypoxia and found that the fALFF values of the bilateral superior temporal gyrus and the right superior frontal gyrus of pilots decreased after hypoxia, whereas the fALFF values of the left precuneus increased. Changes in cognitive function after hypoxia exposure may be associated with brain function. Xu, Chen et al. [5–7], conducted a series of functional imaging studies of pilots’ brains in the resting and task states. Regarding structural magnetic resonance imaging (sMRI) research on pilots, a study conducted by Adamson MM studied the relationship between pilot flight training and the volume of the hippocampus [8]. Cao Yuan et al. [9] explored the age-associated changes in the structure of the brain and its regions in civil aviation pilots, focusing on three aspects: cerebral cortex thickness, surface area and volume. Additionally, Wang Zhenzhen et al. [10] studied the changes in gray matter volume in pilots after accidents. After an accident, the volume of gray matter in some regions of a pilot’s brain was reduced, and half a year later, the gray matter volume of several regions increased again, mainly in the areas that had lost gray matter before. These studies show that long-term flight training and flight experience may have a certain impact on the structure and brain function of pilots’ brains, but no relevant quantitative studies have been carried out.

This study investigated pilots’ brains imaging using sMRI. The experiment collected 3D structural scans using a T1 sequence with the conventional parameters; T1 imaging was chosen to analyze the cerebral cortex structure of pilots because of its high resolution and high contrast. The T1 value is inversely proportional to the longitudinal relaxation time, and the smaller the T1 value is, the stronger the magnetic resonance signal. VBM is a data-driven method which automatically processes and calculates 3D structure voxel by voxel based on T1. VBM segment T1 into gray matter (GM), white matter (WM) and cerebrospinal fluid (CSF). There are many factors that affect the structure of the human brain. Long-term flight training or flight experience is strongly suspected to may have an impact on pilots’ brain structure. Based on pilots’ special working environment and existing research results, it is hypothesized that the vision-related brain regions, being crucial for the core competence of a pilot, may undergo certain structural changes.

The paper mainly introduce the experimental materials, methods, results, discussion and conclusion. The study locates the brain regions where the brain structure is different between pilots and ordinary occupations on T1 structural imaging. Besides, we analyzed the correlation between gray matter volume and flight hours a pilot has completed.

## Materials and methods

### Statement

The data from this study are available upon request. This is because there are legal restrictions. Sharing the physiological data of pilots publicly was forbidden by Civil Aviation Administration of China. Researchers who would like to access the data may contact: kyjd@cafuc.edu.cn (Scientific Research Center of Civil Aviation Flight university of China).

### Participants

Participants recruited were divided into an experimental group composed of pilots and a control group composed of ordinary professionals. All individuals in the experimental group were males ranged from 23–35 years and it contained 26 persons. The pilots had 200–9800 hours of flight time per person, and all of them hold bachelor’s degrees. Their occupations include flight instructors at Civil Aviation Flight University of China, transport aviation pilots, and general aviation pilots. The control group consists of 24 workers in other occupations, matched with the experimental group by age, sex, handedness, and education characteristics. Individuals with a history of neurological illness, traumatic brain injury, substance-related disorders, or standard contraindications to MRI were excluded from this study. The experimental procedures (No. 2018–042002) were approved by the Ethics Committee of the University of Electronic Science and Technology of China (Chengdu, China), and written informed consent was obtained from all participants.

### sMRI data acquisition

The sMRI data were acquired using a 3-T MRI scanner (DISCOVERY MR 750, GE Healthcare, Waukesha WI, United States) at the Center for Information in Medicine of the University of Electronic Science and Technology of China. High-spatial-resolution structural images were acquired using a T1 spoiled gradient-recalled echo pulse sequence. The scan parameters were as follows: repetition time (TR), 5.976 ms; echo time (TE), 1.976 ms, flip angle, 9°; matrix, 256×256; slice thickness, 1 mm (no gap), field of view (FOV), 25.6 cm×25.6 cm, and slice number, 154. Before entering the scanning room, each subject was required to remove all metal objects from the body and wear noise-proof earplugs. Subjects were scanned in a supine, head-first position. Cushions were placed on both sides of the head to decrease head motion. During the scan, all subjects rested in a recumbent position with their eyes closed to eliminate the impact of specific thoughts on the subjects’ brain activity.

### sMRI data preprocessing

Voxel-based morphometry (VBM) [11] is a method that automatically processes and calculates 3D structure voxel by voxel based on T1. It can calculate the density and volume of the whole brain, make quantitative comparisons, and effectively eliminate influence caused by the subjective interpretations of researchers. Accordingly, it has the notable characteristics of automaticity, comprehensiveness and objectivity. VBM is a data-driven method and adopts the diffeomorphic anatomical registration through exponentiated Lie algebra (DARTEL) [12] algorithm, which is a standardized registration method to perform quantitative segmentation with improved accuracy. The T1 is segmented into gray matter (GM), white matter (WM) and cerebrospinal fluid (CSF). Also, the voxels are calculated one by one, which increases accuracy and allows the identification of changes in brain morphology. Through the process, VBM overcomes several drawbacks of morphological measurement based on regions of interest (ROIs): its time requirements, subjectivity, and inability to perform whole-brain analysis.

In this research FSL [13] software was used to process sMRI data. FSL is a comprehensive analysis tool library widely used to process brain fMRI, sMRI and diffusion tensor imaging (DTI) data [14–16]. Most tools in this library can be run from the command line or a graphical interface. Before being processed, the data must be prepared: the raw DICOM image data from the magnetic resonance scanner are converted using the scanning software dcm2nii. The converted data were processed in three steps using FSL software. Firstly, after format conversion, remove non-brain tissues from the images using FSL. Secondly, a template was produced by segmenting the brain tissue into gray matter, white matter and cerebrospinal fluid. The data from randomly selected subjects (40 persons in total) were subjected to image registration by affine transformation (a form of spatial standardization) to the ICBM152 [17]. The transformed data were averaged. Through affine transformation, any pixel X of an image (in this case, a three-dimensional image) can be projected to pixel Y in another space to realize image-matching registration [18], as shown in Formula (1):

Y=MX+T (1)

M is the transformation matrix, T is the translation vector, and X and Y both use three-dimensional coordinates to describe the position of the point in space, as in Formula (2):

$$X=\left\{\begin{array}{c} x_{1}\\ x_{2}\\ x_{3} \end{array}\right\},Y=\left\{\begin{array}{c} y_{1}\\ y_{2}\\ y_{3} \end{array}\right\},M=\left\{\begin{array}{ccc} m_{11} & m_{12} & m_{13}\\ m_{21} & m_{22} & m_{23}\\ m_{31} & m_{32} & m_{33} \end{array}\right\},T=\left\{\begin{array}{c} t_{1}\\ t_{2}\\ t_{3} \end{array}\right\}$$ (2)

**Fig 1:**
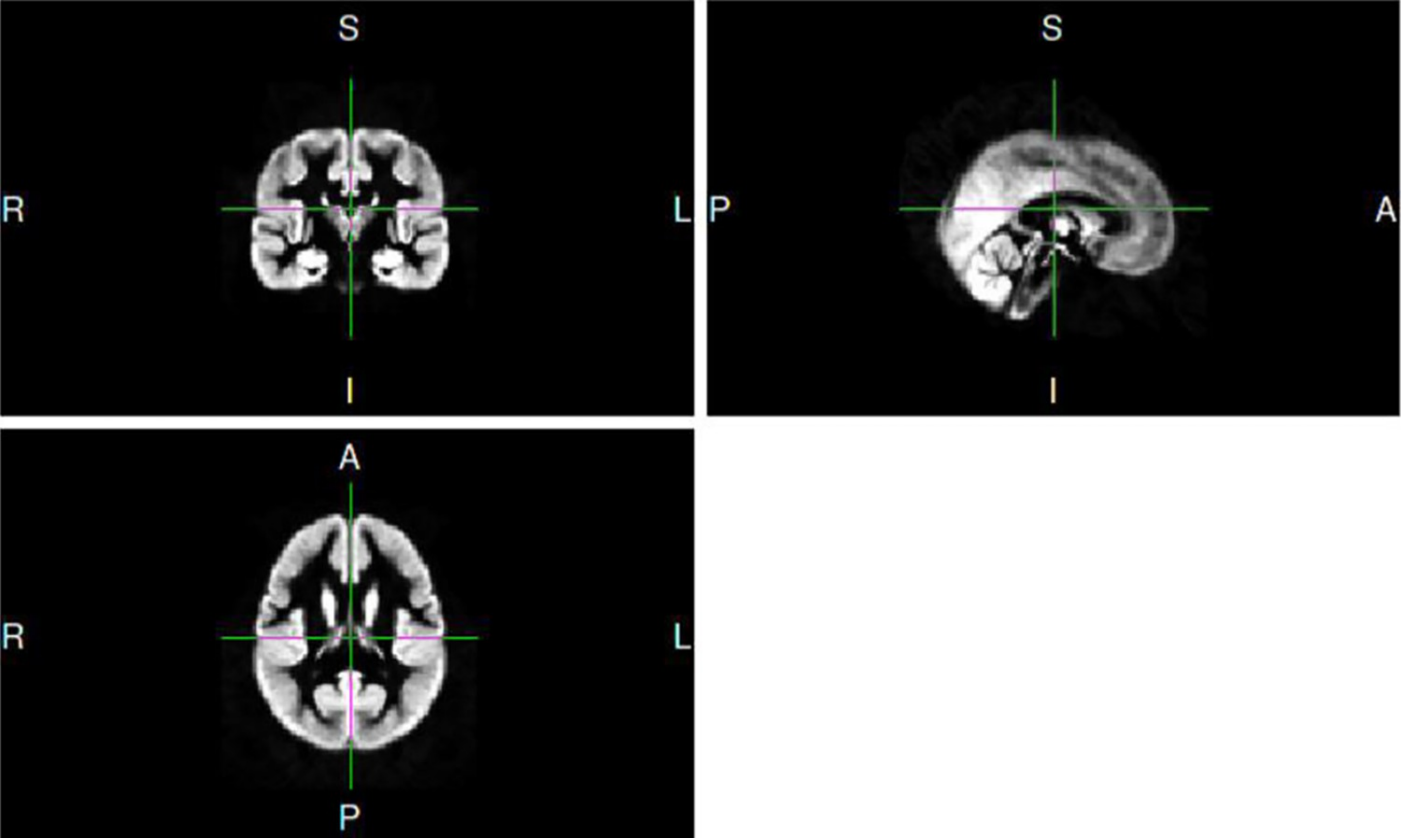
The final gray matter template.

The registered template was flipped along the X axis and added to the unflipped template to obtain a preliminary gray matter template. Then, the subjects who were selected for the template were registered to the preliminary gray matter template through nonlinear transformation. And this template was flipped along the X axis and added to the unflipped template to obtain the final gray matter template after undergoing nonlinear transformation. The template is shown in Fig 1. Thirdly, all participants were registered to the final gray matter template through nonlinear transformation. Also, the Jacobian determinant of the resulting gray matter density image was modulated, and the value of all voxel in this area represents the local absolute gray matter volume. Finally, the modulated image was smoothed with a Gaussian kernel with a full width at half maximum (FWHM) of 3 mm to remove the influence of noise and increase the effectiveness of the statistical tests.

### sMRI statistic analysis

The processed data were statistically analyzed in a Linux environment using the command Randomise [19]. The age of the subjects was set as a covariate, and the permutation test was performed between the experiment group and the control group. The number of permutations was 5000, and the different brain areas were located through statistical analysis. Then, threshold-free cluster enhancement (TFCE) [20] was used to correct for multiple comparisons between the gray matter volumes of the two groups, and significant differences were considered to be present in the brain regions with (P<0.05). The average gray matter volumes of the different brain regions in pilots were extracted. And we remove the extreme values of gray matter volume and number of flight hours that were more than ±3 ×standard deviations from the mean to ensure that the data were normal distribution. Besides, SPSS26 software was used to perform correlation analysis. Pearson correlation analysis was performed on the gray matter volume and flight hours of the experiment group, and the correlation coefficient was calculated.

## Results

### Participants

The 26 pilots and 24 healthy controls were included in the final analysis. Table 1 shows the demographic parameters for the analysis of the difference in gray matter volume.

**Table 1:** Demographic characteristics.

	Pilots (N = 26)		Control (N = 24)		Significance	
	M	SD	M	SD	t value	P value (two-tailed)
Age (years)	25.85	3.08	29.00	4.03	-3.12	0.003*
Sex (% male)	100%		100%			
Education (years)	16		16			
Right-handed (%)	100		100			
Total flight hours	1233.44	2390.04				

M: mean value; SD: standard deviation

### Differences between groups in gray matter volume

**Fig 2:**
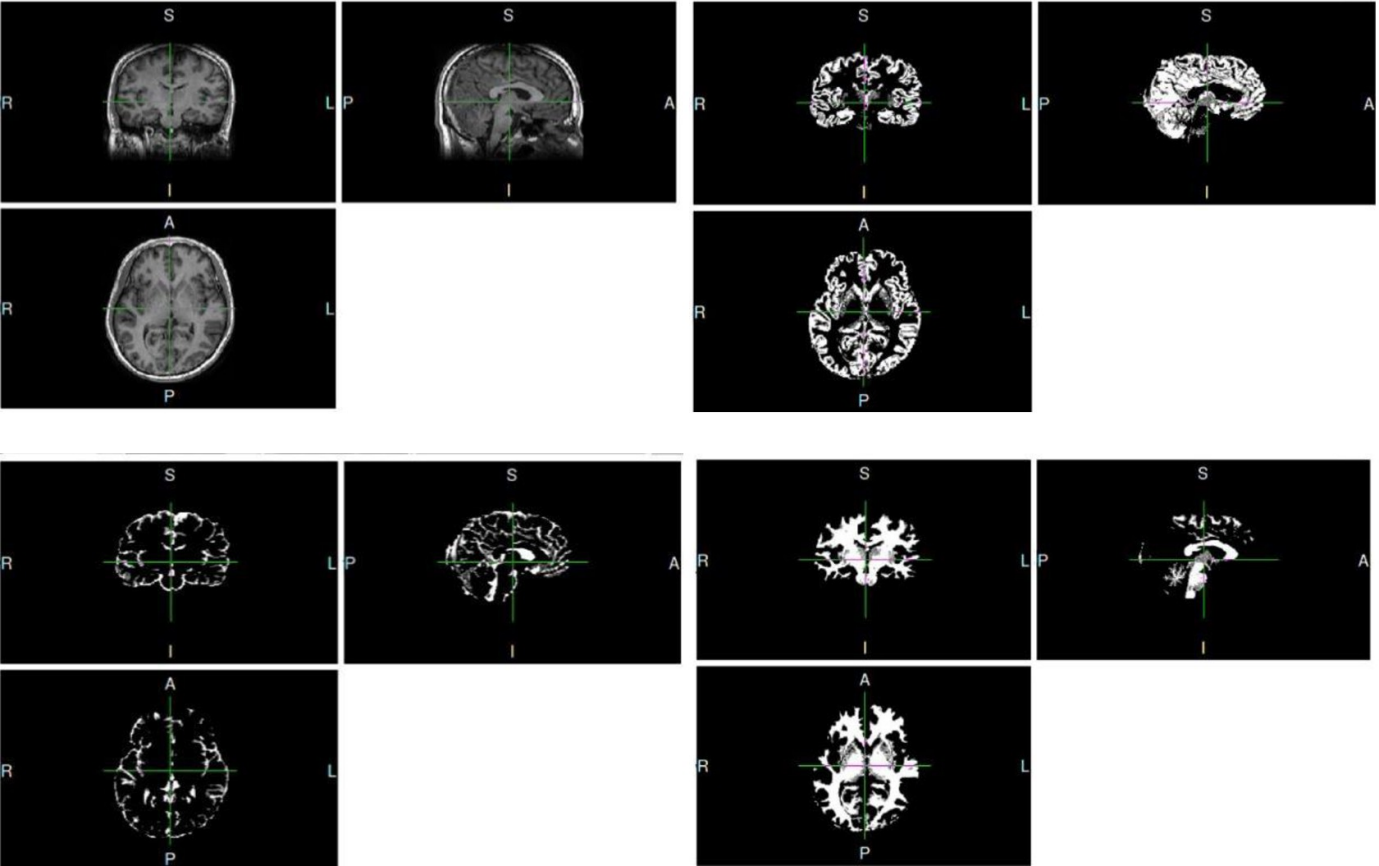
The T1 data (upper left) were segmented into gray matter (upper right), cerebrospinal fluid (lower left), and white matter (lower right).

**Fig 3:**
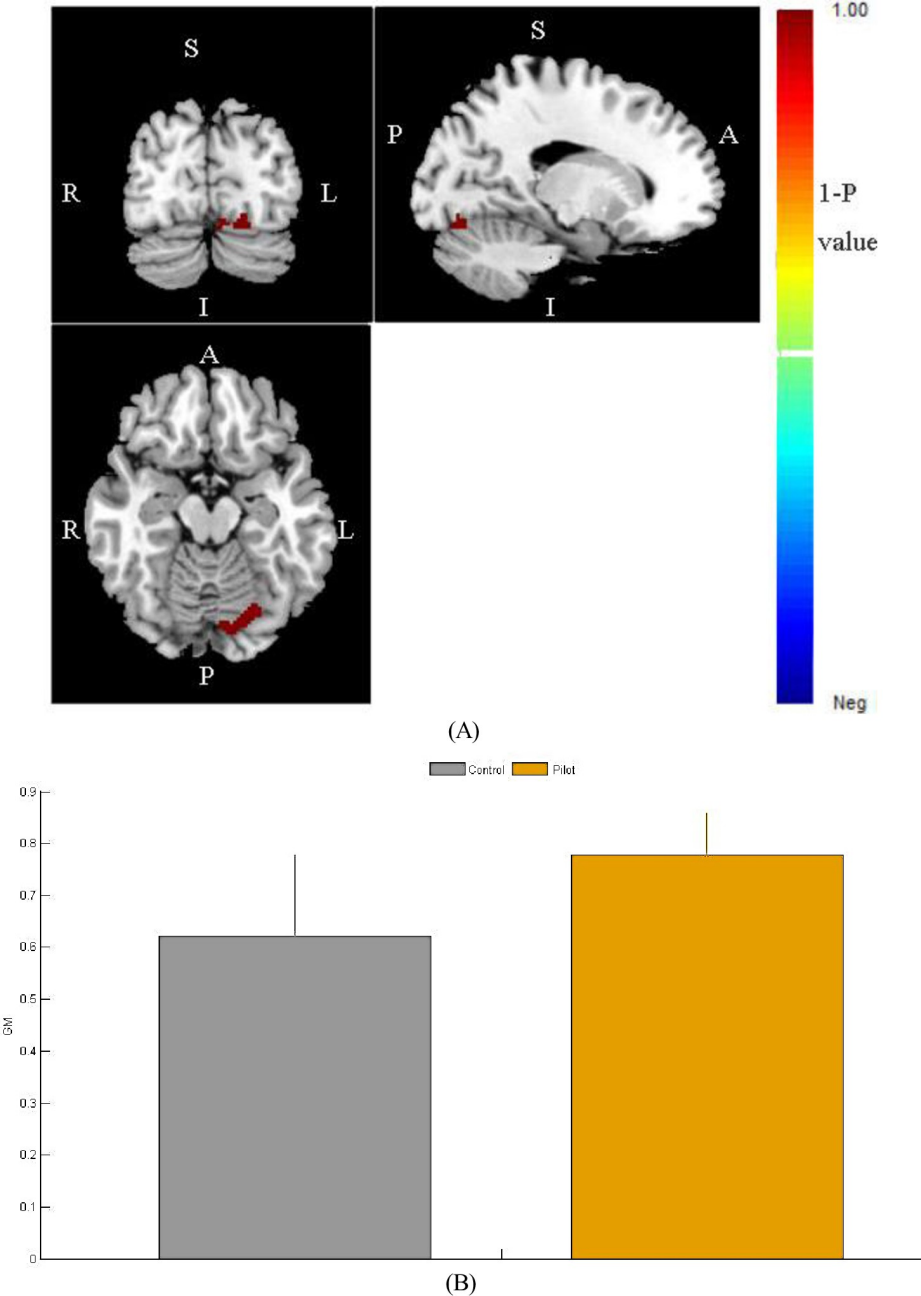
Brain regions with differences and comparison of gray matter volume between the two groups.

The study included pilots and ordinary occupations (control group) as subjects. All subjects in both groups were male, and there were no significant differences in the number of education years. The brain structural T1 magnetic resonance images collected in the experiment were segmented into three parts: cerebrospinal fluid, gray matter and white matter. The segmented sMRI images of gray matter, white matter, and cerebrospinal fluid from one subject are displayed as an example in Fig 2. Statistical models were established through commands for intergroup statistics. The results showed that there is a difference in fusiform and lingual gyrus volume in Fig 3(A) and we can further see that this difference was induced by larger volume in the pilot group relative to the control group by Fig 3(B). The different cluster size and location distribution are shown in Table 2. Both the fusiform gyrus and the lingual gyrus are components of the occipital lobe. The occipital lobe mainly affects the function of the brain that are related to visual memory processing and language information processing [21, 22].

**Table 2:** Brain regions showing group differences in gray matter volume.

Brain regions	Coordinates of the point with the highest 1-P value	1−P value cluster size (voxel number)
	x y z	
fusiform gyrus, lingual gyrus 56 25 29 0.975 162		

### Correlation analysis

**Fig 4:**
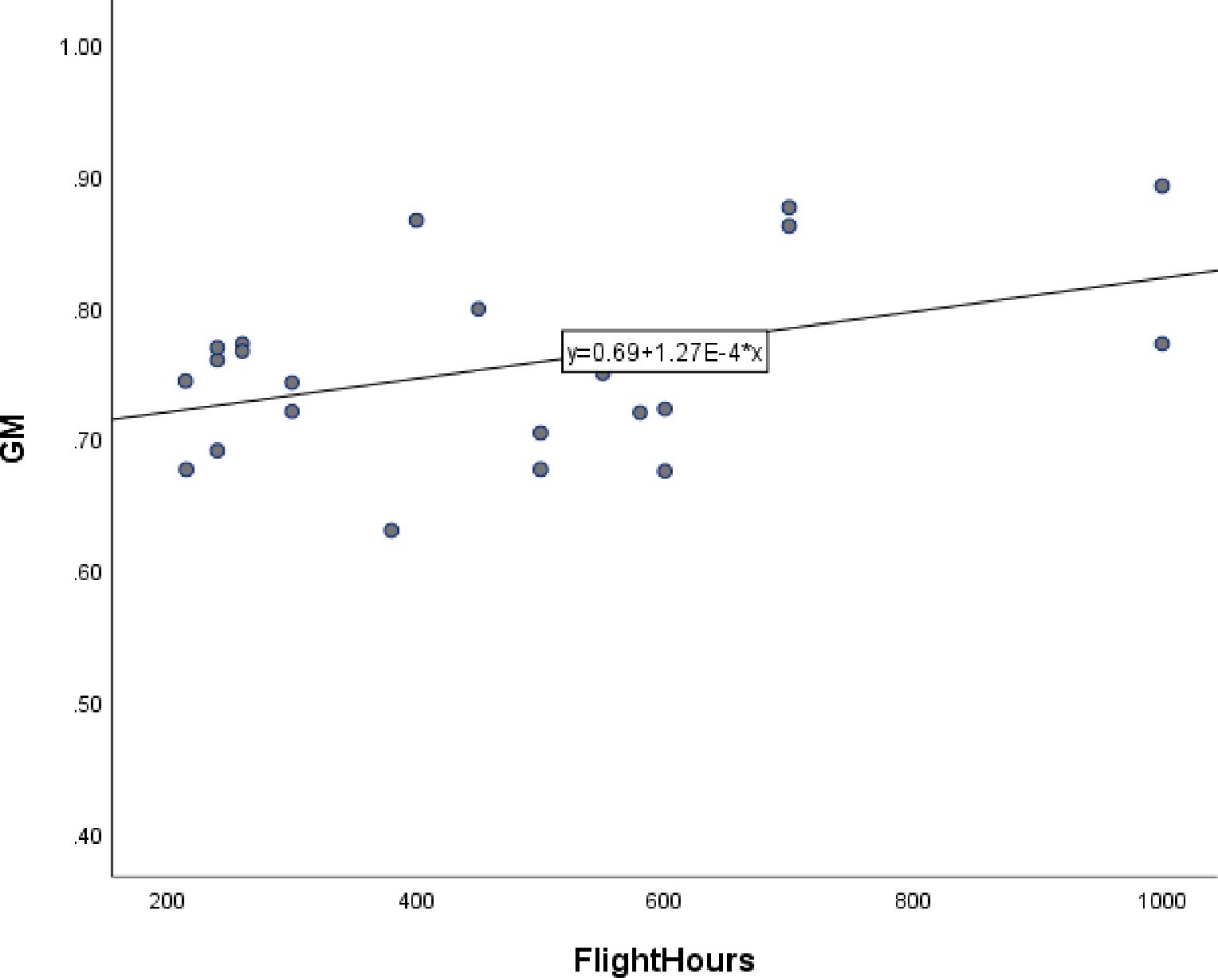
Correlation analysis between pilots’ gray matter volume and flight hours.

The average gray matter volume of the differentiated clusters were evaluated alongside the pilots’ flight hours. Pilots whose gray matter volumes or flight time were more than ±3 standard deviations from the mean were removed from the analysis (4 pilots removed). Then, SPSS26 software was used to analyze the data. First, bar graph of gray matter volume and flight hours of the flight experiment group and the control group were analyzed. Through correlation analysis, a positive correlation was found between the volume of gray matter and the number of flight hours in pilots whose are between 0 and 1000 hours of flight experience (r = 0.426, P = 0.048). Fig 4 shows a plot with the line of best fit. Table 3 shows the demographic parameters for the correlation analysis between the volume of gray matter and the number of flight hours in pilots who had between 0 and 1000 hours of flight experience.

**Table 3:** Demographic characteristics.

	Pilots (N = 22)		Control (N = 24)		Significance	
	M	SD	M	SD	t value	P value (two-tailed)
Age (years)	25.00	1.66	29.00	4.03	-4.464	0.0001*
Sex (% male)	100%		100%			
Education (years)	16		16			
Right-handed (%)	100		100			
Total flight hours	464.98	235.99				

M: mean value; SD: standard deviation

## Discussion

This research employed VBM analysis method to measure and calculate the brain gray matter volume of pilots and ordinary occupations. The results show that the gray matter volumes of the occipital fusiform gyrus and the lingual gyrus were greater in pilots than in the control group. In addition, the gray matter volumes of multiple brain regions in pilots had a positive correlation with the number of flight hours in the initial training phase (r = 0.426, P = 0.048).

Many studies on neural variability have shown that skill acquisition and experience may lead to changes in brain structure and function [23–26]. Previous studies have shown that training causes changes in the volume of gray matter in the cerebral cortex through growth or atrophy of neurons or glia [27]. Most MRI studies in recent years have shown that learning or training can cause the volume of certain brain regions to increase [28, 29]. The changes in gray matter volume are obvious in the early stage of flight training, may because the pilots need to adapt to the training environment and training content. As a result, their flight related functions gradually improve with concurrent increases of the gray matter volume in the lingual gyrus and fusiform gyrus. In previous studies, it was also found that the gray matter volume of pilots who received crisis intervention decreased. But after a period of flight experience, the gray matter volume of some brain regions increased, and there was a great deal of overlap between the brain regions that changed under these two circumstances [30]. With the continuation of flight training and adaptation to the flight environment, we speculate that pilots’ gray matter volume stabilizes along with various functions.

The fusiform gyrus and the lingual gyrus are part of the occipital lobe of the brain. In the human brain, the occipital lobe is relatively small. Because the occipital lobe, at the back of the brain, has three main surfaces: the dorsal lateral surface, the medial surface, and the inferior surface. The part of the occipital lobe between the calcarine fissure and the collateral fissure constitutes the lingual gyrus. The fusiform gyrus is composed of the posterior part between the collateral fissure and the inferior temporal sulcus. The main function of the occipital lobe is to comprehensively process incoming visual information and and it also plays a pivotal role in the connection between the visual system and other sensory systems [21]. It also delivers visual information to the system of brain that perform executive functions [22]. The processing of visual information and the related sensory and executive functions are inseparable from the occipital lobe [31].

Relevant studies have found that the lingual gyrus affects the relationship between visual memory and visual imagery, may because the lingual gyrus is an important area to store visual information [32]. Additionally, another study showed that long-distance semantic connection has significant positive correlations with the imaginative component of creativity and the gray matter volume of the lingual gyrus [33]. Through neuropsychological tests on subjects, it was found that the more comprehensive cognitive function of the lingual gyrus may be attributable to the larger gray matter volume of the lingual gyrus [34]. Further research has found that the cognitive domain of spatial or structural visualization is related to the volume of the lingual gyrus through the retinal nerve fiber layer (RNFL) [35]. The lingual gyrus also has visual processing and word processing functions [36]. In addition, studies have shown that there is a marked positive correlation between the gray matter volume of the left lingual gyrus and an individual’s capacity of sustained attention [37]. In summary, the greater gray matter volume of pilots’ lingual gyrus is probably because pilots need to pay attention to flight conditions for long periods time during flight. Therefore, pilots may have a stronger capacity of continuous attention than ordinary occupations. At the same time, pilots also need to observe the instrumentation in the cockpit and must fully interpret the readings, which may strengthen their visual memory and visual processing capabilities. Moreover, studies have found that the spontaneous activity of neurons in the lingual gyrus is higher in pilots than in ordinary professionals [38].

The fusiform gyrus, between the inferior temporal sulcus and the collateral fissure, is mainly responsible for the processing of auditory information. Besides, it is still related to understanding and memory of the language, and may affect people’s memory capacity in daily life [39]. Through experimental research, it has been found that the gray matter volume of the right fusiform gyrus may play a major role in cognitive changes [38]. The volume of the fusiform gyrus is increased in pilots compared to controls, which may because pilots need to communicate vocally with other personnel during flight and thus has strengthened the ability to process and memorize auditory information. Moreover, language organization and expression skills are the key areas of examination for training pilots [40]. The fusiform gyrus has been confirmed to be related to visuospatial function by most studies, and the right side is more involved in this function than the left [36]. Visuospatial ability is usually divided into visuospatial perception ability and visual performance ability. Visuospatial perception ability is divided into the ability to perceive grades of light and darkness, the ability to perceive color, and other abilities [41]. Visual performance ability is divided into detail and spatial performance ability [42]. Pilots have extensive experience working in a cockpit composed of electronic instruments in various directions, and their ability to perceive information on various dials has been enhanced compared to that of non-pilots. Additionally, to facilitate distinction, the various instrument outputs in the cockpit have different color designs, and pilots must respond to changes of the information in a timely manner. So pilots’ perception of some colors may be enhanced.

This study still has some shortcomings. First, the sample size was small; the experimental results need to be verified in a larger sample. Second, age differences cannot be completely excluded as a factor influencing the results. Third, this study lacked relevant tests to measure visual-process ability. Follow-up research will increase the sample size and collect experimental data longitudinally from pilots to explore the characteristics of brain changes. The goals of such research will explain the differences in brain functional mechanisms between extensively trained pilots and ordinary occupations on the basis of imaging, identify the brain areas that support strengthened occupation-related brain functions in pilots, and provide an objective brain imaging basis for the evaluation and selection of flight training.

## Conclusion

In this study, T1 structural MRI was used to analyze the structural differences in the gray matter of the brain between pilots and ordinary occupations. The results showed that the volumes of gray matter in the fusiform gyrus of the occipital lobe and the lingual gyrus are increased in the brains of pilots. It is possible that the pilots’ long-term flight experience cause their brain structure to change. In terms of visual processing and further optimization, visuospatial function maybe enhanced in pilots. In addition, pilots may have an enhanced ability to pay sustained attention. Occupational changes in pilots’ brain structure progress most rapidly in the early training period; with the continuation of flight training and adaptation to the flight environment, pilots’ functions and gray matter volume stabilize. These research results show that increased gray matter volume maybe beneficial to pilots’ visual function during flight. The results also highlight gray matter volume as a quantitative feature of brain structure that could be used to identify high-quality flight training.
